# Hyaluronic acid-coated chitosan nanoparticles induce ROS-mediated tumor cell apoptosis and enhance antitumor efficiency by targeted drug delivery via CD44

**DOI:** 10.1186/s12951-016-0245-2

**Published:** 2017-01-10

**Authors:** Tao Wang, Jiahui Hou, Chang Su, Liang Zhao, Yijie Shi

**Affiliations:** 1 School of Pharmacy, Jinzhou Medical University, Jinzhou, 121000 People’s Republic of China; 2 School of Veterinary Medicine, Jinzhou Medical University, Jinzhou, 121000 People’s Republic of China

**Keywords:** Nanoparticles, Hyaluronic acid, CD44, Mitochondria, Reactive oxygen species

## Abstract

**Background:**

A targeted drug nanoparticle (NP) delivery system has shown potential as a possible cancer treatment. Given its merits, such as its selective distribution at tumor sites and its controllable drug release, drug-loaded NPs can be effectively delivered to selected organs and targeted cells, thus enhancing its antitumor efficiency and reducing its toxicity.

**Methods:**

We reported that hyaluronic acid (HA)-coated chitosan NPs promoted the drug delivery of 5-fluorouracil (5-Fu) into tumor cells that highly expressed CD44.

**Results:**

Our new findings suggested that HA-coated chitosan NPs enhanced drug accumulation by effectively transporting NPs into CD44-overexpressed tumor cells, and they also resulted in mitochondrial damage induced by the production of reactive oxygen species (ROS). Compared to free drug and uncoated NPs, HA-coated chitosan NPs exhibited stronger inhibition rates and induced obvious apoptosis in CD44-overexpressed A549 cells.

**Conclusions:**

Biocompatible and biodegradable HA-coated chitosan NPs were developed to encapsulate a chemotherapeutic drug (5-Fu) to enhance drug accumulation in tumor cells and to improve the agent’s antitumor efficiency by offering targeted drug delivery via CD44.

## Background

Current cancer treatment primarily depends on surgical operations and chemotherapy to fight against cancer. Although chemotherapeutic drugs are effective at killing tumor cells, some have several drawbacks, including their nonselective distribution, drug toxicity, and unexpected side effects to normal tissues, which have limited these agents’ clinical use [1–3]. There were few effective anti-cancer drugs available that would specifically kill cancer cells and not damage normal cells. Therefore, the onset of side effects and toxicity induced by the anti-tumor drugs was probably inevitable and resulted in the failure of chemotherapy. CD44 is a multistructural and multifunctional cell-surface molecule involved in cell proliferation, cell differentiation, cell migration, and angiogenesis, and it is also implicated in cell signaling for survival [4–7]. Compared with normal cells, CD44 showed a higher expression level in many cancer cells and was recognized as a potential therapeutic target in cancer therapy [8, 9]. Hyaluronic acid (HA) is a type of linear mucopolysaccharide composed of alternately repeated N-acetylglucosamine and glucuronic di-saccharide, and it constitutes the main component of the extracellular matrix. Owing to its ability to specifically target CD44 receptors, HA has been frequently modified with a drug carrier to enhance drug delivery in CD44-overexpressed tumor cells to effectively inhibit tumor growth [10–15].

Nanoparticles (NPs), an effective drug delivery system, have shown potential clinical application in the treatment of cancer. In recent years, numerous studies confirmed the role of NPs in enhancing the accumulation of drugs at the tumor site and controlling the drug release rate to prolong curing times [16–18]. Furthermore, related studies showed how the internalization of some NPs increased the generation of reactive oxygen species (ROS) and enhanced synergistic antitumor efficacy by activating mitochondria-meditated apoptosis [19–22]. To investigate whether drug-loaded NPs could induce more cell apoptosis, and to further determine whether NPs activated the ROS-mediated mitochondrial apoptosis pathway, we designed HA-coated chitosan (CS) NPs to enhance antitumor efficiency via targeted drug delivery by way of the interactions between HA and CD44. Then, 5-fluorouracil (5-Fu), as a model anticancer drug, was used to prepare 5-Fu-loaded HA-coated CS NPs by the ion gelation method, and the loading efficiency (LE), encapsulation efficiency (EE), and drug release process were also explored in vitro. The cellular uptake and distribution of HA-coated NPs were investigated in A549 cells (which overexpress CD44 receptors) and HepG2 cells (those that feature low-expressing CD44 receptors) to evaluate NPs’ ability to specifically target the CD44 receptors. Cell cytotoxicity and cellular apoptosis were assessed to confirm ROS-mediated mitochondrial apoptosis and to evaluate the HA-coated CS NPs’ antitumor efficiency.

## Methods

### Materials

CS, with a degree of deacetylation of 80% and a molecular weight of approximately 400 kDa, was purchased from Haixin Biological Product Co., Ltd. (Ningbo, People’s Republic of China); 3-(4,5-dimethylthiazol-2-yl)-2,5-diphenyltetrazolium bromide (MTT) and rhodamine B (RhB) were purchased from Sigma-Aldrich Co. (St Louis, MO, USA); sodium hyaluronate (molecular weight: 125 kDa) was obtained from Shandong Freda Biochem Co, Ltd (Shandong, People’s Republic of China); and 5-Fu was purchased from Nantong Jinghua Pharmaceutical Co, Ltd. (Nantong, People’s Republic of China). The other chemicals that were purchased were of analytical grade and were obtained from Sigma-Aldrich Co. A549 cells and HepG2 cells were obtained from Jinzhou Medical University (Jinzhou, People’s Republic of China).

### Preparation of 5-Fu-loaded HA-coated CS NPs

According to a previous protocol [23, 24], we first prepared 5-Fu-loaded CS NPs using the ion gelation method. To prepare 5-Fu-loaded NPs, 1 mg of 5-Fu was added into a solution containing 500 mL of acetic acid (2%, v/v) and 20 mg of CS, and the obtained sodium tripolyphosphate reserve liquid was added into the CS solution by slow dropping under magnetic stirring for 5 h at room temperature. Finally, 5-Fu-loaded CS NPs formed instantaneously and the NPs were precipitated and resuspended in distilled water, followed by the addition of HA sodium salt solution under oscillation for 30 min. Owing to the strong electrostatic interaction between the cationic amino group of CS and the anionic carboxyl group of HA, HA was conjugated at the surface of the CS NPs by charge adsorption to obtain 5-Fu-loaded HA-coated CS NPs. The obtained 5-Fu loaded HA coated CS NPs were precipitated by centrifugation at 16,000 rpm for 20 min, then the NPs were separated and washed with distilled water for three times to remove the free HA and chitosan. The structure of HA-coated CS NPs was investigated using IRAffinity-1 infrared (IR) spectroscopy, and the physical characteristics of NPs including their morphology, particle size, zeta potential, and polydispersity index were determined by transmission electron microscope (TEM) (JEM-1200EX; JEOL, Tokyo, Japan) and Zetasizer (Nano ZS90; Malvern Instruments, Malvern, UK). The LE, EE, and the in vitro drug-release process were also explored [25].

### MTT

The cytotoxicity of free 5-Fu and 5-Fu-loaded NPs of different concentrations were evaluated by MTT assay. Cells (80 μL per well, about 1 × 10^4^ cells/well) were cultured in clear-bottom 96-well tissue culture plates. Test groups including free 5-Fu, 5-Fu-loaded HA-coated CS NPs, and a combination of free HA and 5-Fu-loaded HA-coated CS NPs were added and the cells were incubated for 48 h. The culture medium was removed and 100 μL of Dulbecco’s Modified Eagle’s Medium (DMEM) was added. Then, 15 μL of MTT with a concentration of 5 mg/mL was added into the wells and incubated with the cells for 4 h at 37 °C. Finally, 150 μL of dimethyl sulfoxide (DMSO) was added into each well followed by the gentle mixing on an orbital shaker for 1 h at room temperature. The absorbance was measured at 570 nm for each well by an absorbance microplate reader (Synery-2; BioTek Instruments, Winooski, VT, USA). All experiments were performed in triplicate.

### In vitro cellular uptake

In order to observe the intracellular distribution of NPs, RhB as a fluorescent marker was encapsulated in NPs to label their location within the cells. RhB-labeled HA-coated CS NPs were incubated with cells in the medium containing free HA or no HA for a certain period of time. The nucleus was stained with Hoechst (blue) for 15 min at 37 °C and the mitochondria were stained by Mito tracker (green). Finally, the internalizing process of RhB-labeled NPs was observed by detecting the fluorescence of RhoB (Ex:572 nm, Em: 591 nm), hoechst33342 (Ex:352 nm, Em: 455 nm) and Mito tracker (Ex:495 nm, Em: 519 nm) when culturing the cells on the built-in culture stage of confocal laser scanning microscopy (CLSM) (FluoView FV10i; Olympus Corporation, Tokyo, Japan) at 37 °C under 5% CO_2_ and humidity control, enabling long-term live cell imaging. For cell culture, a glass bottom dish, 35 × 10 mm, advanced TC (treated) was set on the stage incubator. On the detection side,the FV10i was fitted with a newly developed spectral system featuring two fluorescence channels supplied by a novel grating, beam splitter and slit arrangement. In addition, each channel was fitted with a variable barrier filter which was set automatically to match the wavelength range for each fluorescence dye in use.

### JC-1 staining

The mitochondrial membrane potential change was measured using JC-1 (Beyotime Institute of Biotechnology, Haimen, People’s Republic of China) and it was observed under CLSM (FluoView FV10i; Olympus Corporation). Briefly, A549 cells (1 × 10^5^) were seeded in a glass-bottomed dish (NEST, φ15 mm; People’s Republic of China) and cultured for 24 h under 5% CO_2_ at 37 °C. After that, the cells were treated with fresh medium containing 5-Fu, 5-Fu-loaded HA-coated CS NPs, and a combination of free HA and 5-Fu-loaded HA-coated CS NPs, respectively, for another 24 h. After incubation, the medium was removed and the cells were washed twice with cold phosphate buffered saline (PBS); the cells were then stained with JC-1 (5 mg/mL) at 37 °C for 30 min in the dark. Stained cells were washed twice with cold PBS to remove free dye and they were observed by CLSM.

### ROS determination and endoplasmic reticulum stress

To investigate the effects of NP exposure on the production of ROS, as well as on the function of both endoplasmic reticulum (ER) stress and the mitochondria, mitochondrial permeability was detected after NP exposure using the JC-1 staining method; the degree of ER stress was also evaluated by observing the morphological changes of the ER under CLSM. The intracellular DCF fluorescence intensity, which is excited at 485 nm and emitted at 530 nm, was detected using a microplate reader (Synery-2; BioTek Instruments) to investigate the extent of oxidative stress. To examine the role of NPs on ROS-induced mitochondrial disorders after NP exposure, N-acetyl-l-cysteine (NAC), an ROS inhibitor, was induced to determine the vital effect of oxidative stress on mitochondrial morphology dysfunction induced by NP exposure.

### Cell apoptosis study

The apoptotic effects of 5-Fu-loaded HA-coated CS NPs on CD44 overexpressed A549 cells were determined using the Annexin V-FITC Apoptosis Detection Kit (Abcam plc, Cambridge, UK). Tested cells were seeded in 6-well plates (Corning, Inc., Corning, NY, USA) at a density of 1 × 10^5^ cells/well in 1 mL of medium. After 24 h, the cells were treated, respectively, with fresh medium containing 5-Fu, 5-Fu-loaded HA-coated CS NPs, and the combination of free HA and 5-Fu-loaded HA-coated CS NPs. After 24 h of treatment at 37 °C with 5% CO_2_, cells were digested with 0.25% trypsin and washed twice with cold PBS. The collected cells were stained with Annexin V-FITC and propidium iodide (PI; provided in the kit) in the dark and analyzed by flow cytometry (BD, Franklin Lakes, NJ, USA).

### Western blot assay

The related protein expressions in A549 cells treated with various formulations were detected by means of Western blot analysis. A549 cells were harvested and lysated in radioimmunoprecipitation assay (RIPA) buffer (150 mM of NaCl, 1% NP-40, 1% sodium dodecyl sulfate (SDS), 1 mM Phenylmethanesulfonyl fluoride (PMSF), 10 ug/mL of leupeptin, 1 mM of aprotinin, 50 mM of Tris–Cl, pH 7.4). The cells were incubated in ice for 30 min and lysates were centrifuged for 20 min at 13,000 rpm to acquire the supernatant. The protein concentration was determined by the BCA Protein Assay Kit (Beyotime Institute of Biotechnology). The protein lysates were separated by 12% SDS–polyacrylamide gel electrophoresis (PAGE) and transferred to polyvinylidene fluoride (PVDF) membranes (BioTrace; Pall Corporation, New York, USA). After blocking with 1% bovine serum albumin (BSA) for 1 h at room temperature, membranes were incubated with 1:1000 diluted primary antibody overnight at 4 °C. Then, the membranes were washed and incubated with 1:10,000 diluted secondary antibodies for 1 h at room temperature. The membranes were washed again and stained with enhanced chemiluminescence (ECL). The protein bands were visualized by ECL detection reagents and captured by the Bio-RAD Gel Imaging System.

## Results

### The preparation and characteristics of various kinds of NPs

**Fig. 1:**
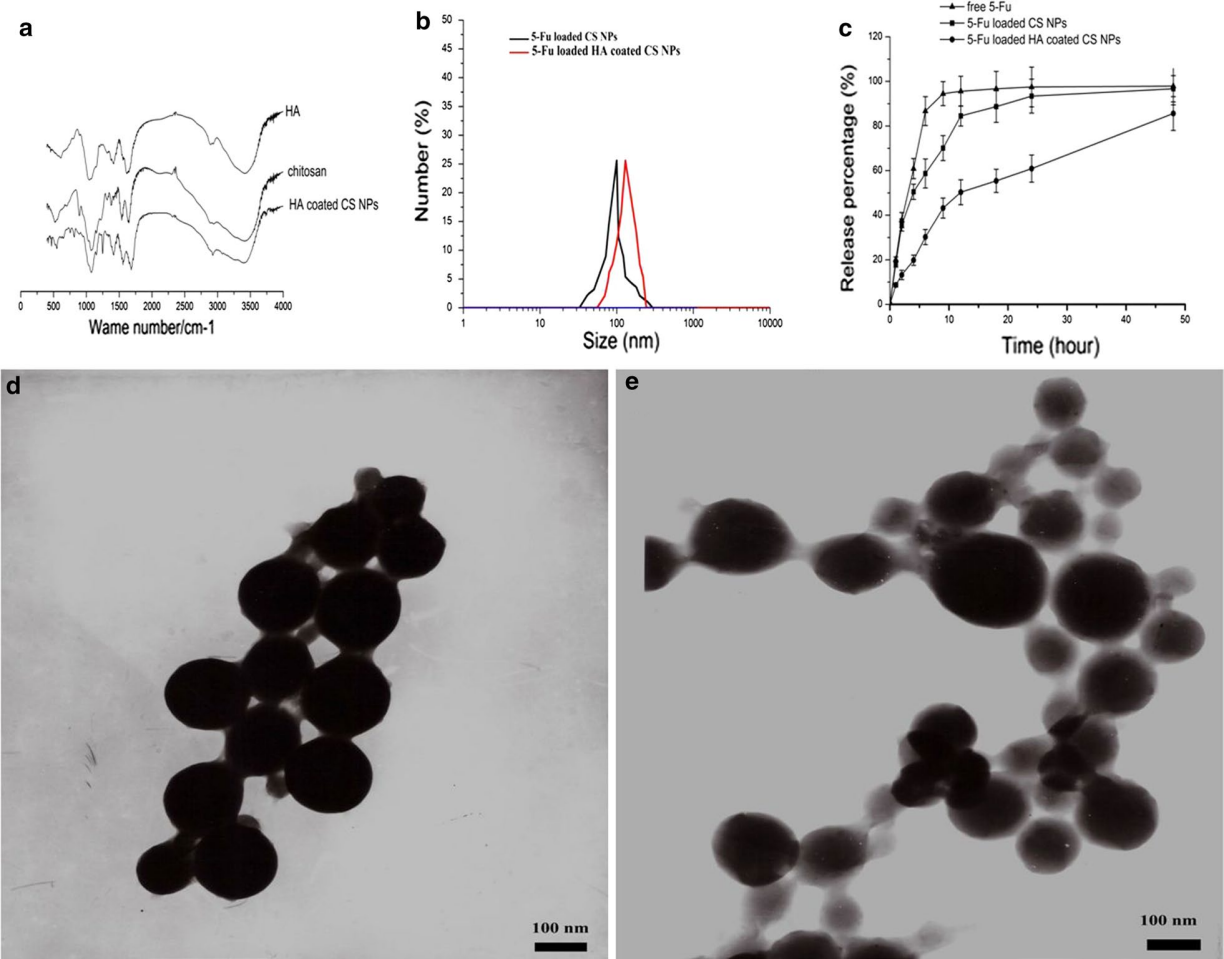
Characterization of 5-Fu-loaded HA-coated CS NPs. **a** FTIR spectra of chitosan, HA and HA-coated CS NPs; **b** DLS analysis of 5-Fu-loaded HA-coated CS NPs; **c** accumulated release of drug-loaded NPs in the medium at pH 7.4; and TEM images of 5-Fu-loaded HA-coated CS NPs (**d**) and 5-Fu-loaded CS NPs (**e**). The results are expressed as the mean ± SD (n = 3)

The construction of HA-coated CS NPs was investigated using IR spectroscopy, as shown in Fig. 1a. It was found that the characteristic absorption peaks of CS and HA were all visible along the spectrum of the HA-coated CS NPs. This suggests that CS and HA, as materials, were involved in NP formation. Compared with the spectra of CS and HA, there were no obvious new absorption peaks that appeared along the spectrum of HA-coated CS NPs, thus indicating that no new bonds were formed during the preparation of HA-coated CS NPs. HA absorbed the surface of the CS NPs via electrostatic interactions between CS and HA, rather than by chemical conjugation. The characteristics of various kinds of NPs were investigated to determine their particle size, zeta potential, and morphology. The results in Table 1 show that the size of 5-Fu-loaded HA-coated CS NPs was smaller and more homogeneously distributed; moreover, the morphology of the NPs was subspheroidal and its zeta potential was positive and valued at 15.6 ± 3.7 mV. The average EE of the CS NPs and HA-coated CS NPs was 85.2 ± 5.44% and 86.7 ± 6.18%, respectively, indicating that no drugs were leaking from the CS NPs during the HA coating process. Compared with free drug and 5-Fu-loaded CS NPs, 5-Fu-loaded HA-coated CS NPs showed a sustained and slower biphasic release profile, and over 75% of the total 5-Fu was released at 48 h. This suggests that the coating of HA at the surface of CS NPs may have increased the particle size, and that the medium solution took longer to penetrate through the polymer matrix into the interior of the NPs, thus leading to sustained and smooth drug release.

**Table 1:** Key parameters of 5-Fu loaded CS NPs and 5-Fu loaded HA coated CS NPs

Group	Diameter (nm)	Zeta potential (mV)	Polydispersity	Encapsulation efficiency %
5-Fu loaded CS NPs	98.5 ± 5.4	21.9 ± 2.6	0.31 ± 0.09	85.2 ± 5.44
5-Fu loaded HA coated CS NPs	118.9 ± 9.8	15.6 ± 3.7	0.19 ± 0.05	86.7 ± 6.18

### MTT analysis

**Fig. 2:**
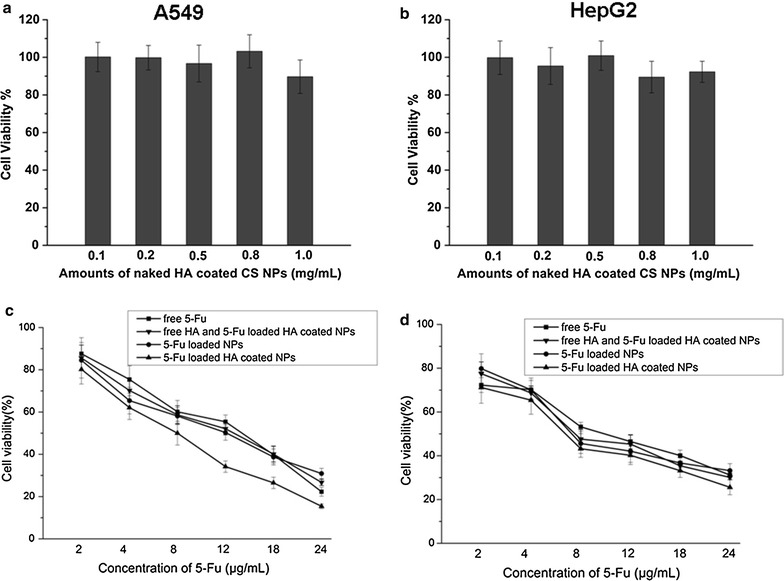
Viability of A549 cells and HepG2 cells. Viability of A549 cells (**a**) and HepG2 cells (**b**) after incubation with naked HA-coated CS NPs. Viability of A549 cells (**c**) and HepG2 cells (**d**) after incubation with free 5-Fu, 5-Fu-loaded CS NPs, 5-Fu-loaded HA-coated CS NPs and the combination of free HA and 5-Fu-loaded HA-coated CS NPs for 48 h. The results are expressed as the mean ± SD (n = 3)

The MTT results shown in Fig. 2 demonstrate that naked HA-coated CS NPs at a certain concentration (ranging from 0.1 to 1.0 mg/mL) did not inhibit the proliferation of A549 cells and HepG2 cells, and that the viability ratios were over 85%, indicating its good excellent biocompatibility and nontoxicity. Compared with the free drug and 5-Fu-loaded uncoated CS NPs, 5-Fu-loaded HA-coated CS NPs showed higher cytotoxicity in both cells. The 50% maximal inhibitory concentration (IC50) values in A549 cells and HepG2 cells treated with 5-Fu-loaded HA-coated CS NPs were 8.0 and 6.7 μg/mL at 48 h, respectively. Interestingly, with the pre-addition of free HA into both cells, cells treated with NPs showed different changing trends in their ability to inhibit the effects of treated cells. With the addition of HA into A549 cells (those overexpressing CD44 receptors), the cytotoxic effects of CS NPs were significantly inhibited and the IC50 value in A549 cells treated with 5-Fu-loaded HA-coated CS NPs was reduced to 13.1 μg/mL at 48 h. Conversely, the addition of free HA had no obvious effects on the HepG2 cells’ (those with low-expressing CD44 receptors) cell proliferation, and the IC50 value in cells treated with 5-Fu-loaded HA-coated CS NPs was 7.2 μg/mL at 48 h. This suggested that in A549 cells (those overexpressing CD44 receptors), HA as a targeting ligand improved the internalization of NPs in those cells that overexpressed the CD44 receptor, and 5-Fu-loaded HA-coated CS NPs showed the highest drug cytotoxicity, as compared with free drugs and drug-uncoated HA NPs. With the mediation of free HA, HA in the medium and 5-Fu-loaded HA-coated CS NPs competed to bind with its CD44 receptor; the uptake of 5-Fu-loaded HA-coated CS NPs was reduced, thus decreasing the intracellular distribution and concentration of drugs, leading to the inhibition of cytotoxicity. This further proved that 5-Fu-loaded HA-coated CS NPs may be internalized via HA-mediated uptake.

### Uptake ability of different kinds of NPs in cells

**Fig. 3:**
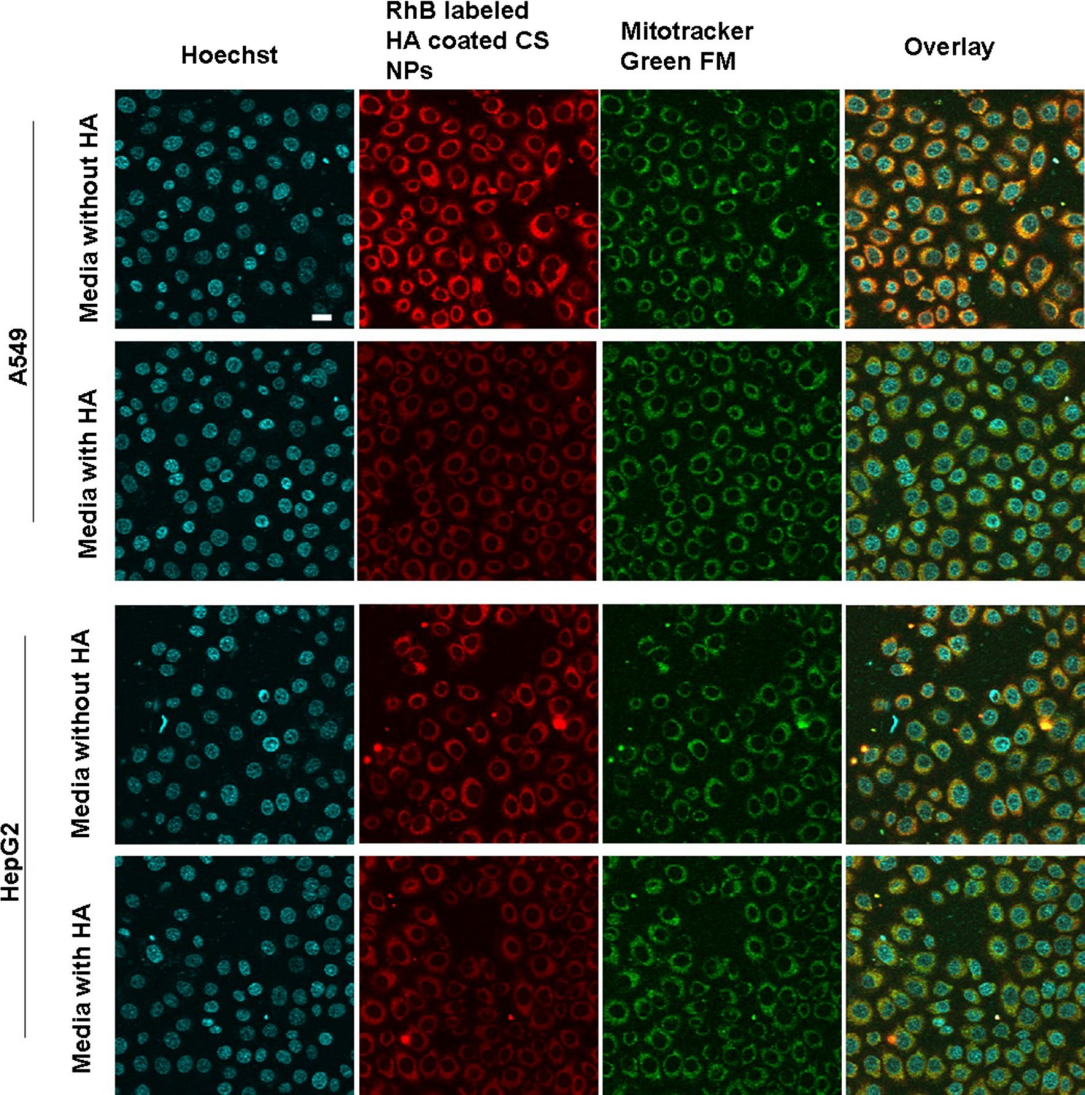
The in vitro cellular distribution of RhB-labeled HA-coated CS NPs following the incubation of A549 cells and HepG2 cells. The *scale bar* in all figure parts is 100 μm

When RhB-labeled HA-coated CS NPs were co-incubated with cells for 6 h (Fig. 3), NPs were first adsorbed on the surface of the cell membrane and they were further internalized into the cell’s interior, represented by the dispersion of intense red fluorescent dots in the cytoplasm. Compared with the weak fluorescent dots observed in HepG2 cells treated with HA-coated CS NPs, stronger and intense red fluorescence emitted by RhB-labeled HA-coated CS NPs appeared in the cytoplasm of A549 cells that highly expressed CD44. This indicated that HA-coated CS NPs effectively promoted drug accumulation in those cancer cells where CD44 was highly expressed on the cell surface; specific binding between HA and CD44 might facilitate the uptake of HA-coated CS NPs. To further clarify the internalization process of HA-coated CS NPs mediated by the interaction between HA and CD44, we used free HA as a competitive inhibitor of CD44 receptors to pre-coincubate the cells to investigate the competitive cellular uptake mechanism of NPs. The results showed that after A549 cells were cultured with HA-coated CS NPs, intense red fluorescence was observed inside the cells. However, following the addition of 2 mg/mL of free HA into the medium, intracellular fluorescent intensity was significantly reduced and the NP uptake was significantly inhibited. This suggested that HA-coated CS NPs selectively bound to CD44 receptors and accelerated the internalization of NPs into cells. Free HA that existed in the medium had competed with HA-coated CS NPs to bind to CD44 receptors on the cell surface, thus resulting in receptor saturation and preventing the transportation of NPs into the cells. In contrast, the addition of HA had no obvious effects on the internalization of HA-coated CS NPs in HepG2 cells with a low expression of CD44. Furthermore, the obvious co-location between NPs and mitochondria was observed, and it was demonstrated that HA-coated NPs were situated at the mitochondria, where they interfered with the integrity and function of the mitochondria. This indicated that mitochondrial-mediated apoptosis may have been triggered.

### JC-1 staining

**Fig. 4:**
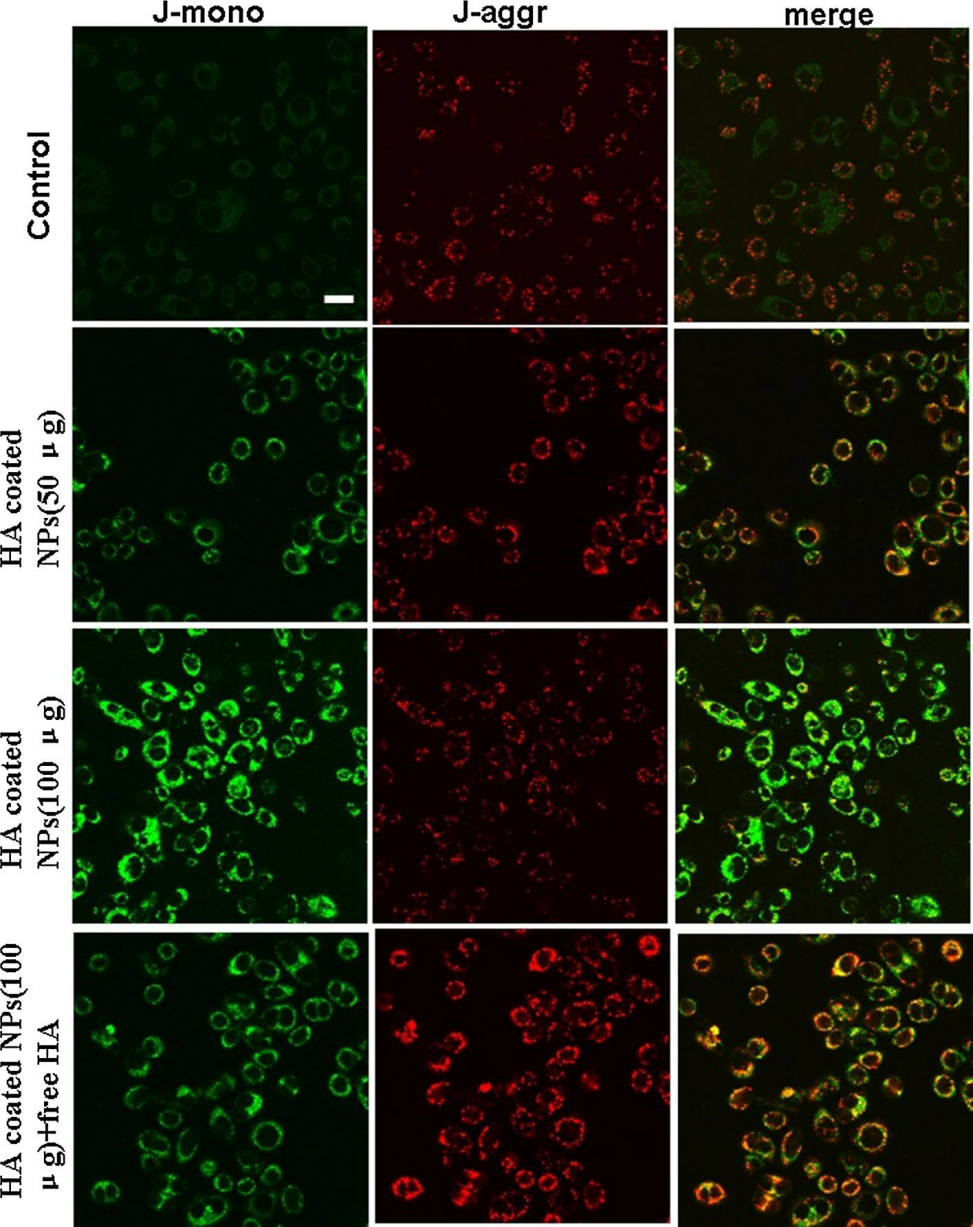
Changes in the mitochondrial membrane potential after incubating HA-coated CS NPs with A549 cells. The *scale bar* in all figure parts is 100 μm

In order to further investigate the effects of HA-coated NPs on the integrity and permeability of mitochondria, JC-1 staining was performed to detect the potential change in the mitochondria by observing the color variations between green fluorescence (JC-1 monomer) and red fluorescence (JC-1 aggregation). As illustrated in Fig. 4, the intensity of the green fluorescence in cells treated with HA-coated CS NPs was significantly enhanced, indicating that HA-coated NPs were located at the mitochondria; these NPs damaged the integrity of the mitochondria, thus causing a significant decrease in the mitochondrial membrane potential. With the increasing addition of HA-coated NPs, the membrane potential continued to decline in a dose-dependent pattern, represented by a proportional decrease in the intensity of both red fluorescence and green fluorescence. Additionally, it was demonstrated that the addition of free HA reduced the accumulation of NPs in the mitochondria, ultimately maintaining the integrity and permeability of the mitochondria through binding competition.

### The effect of NP exposure on ROS generation and ER stress

**Fig. 5:**
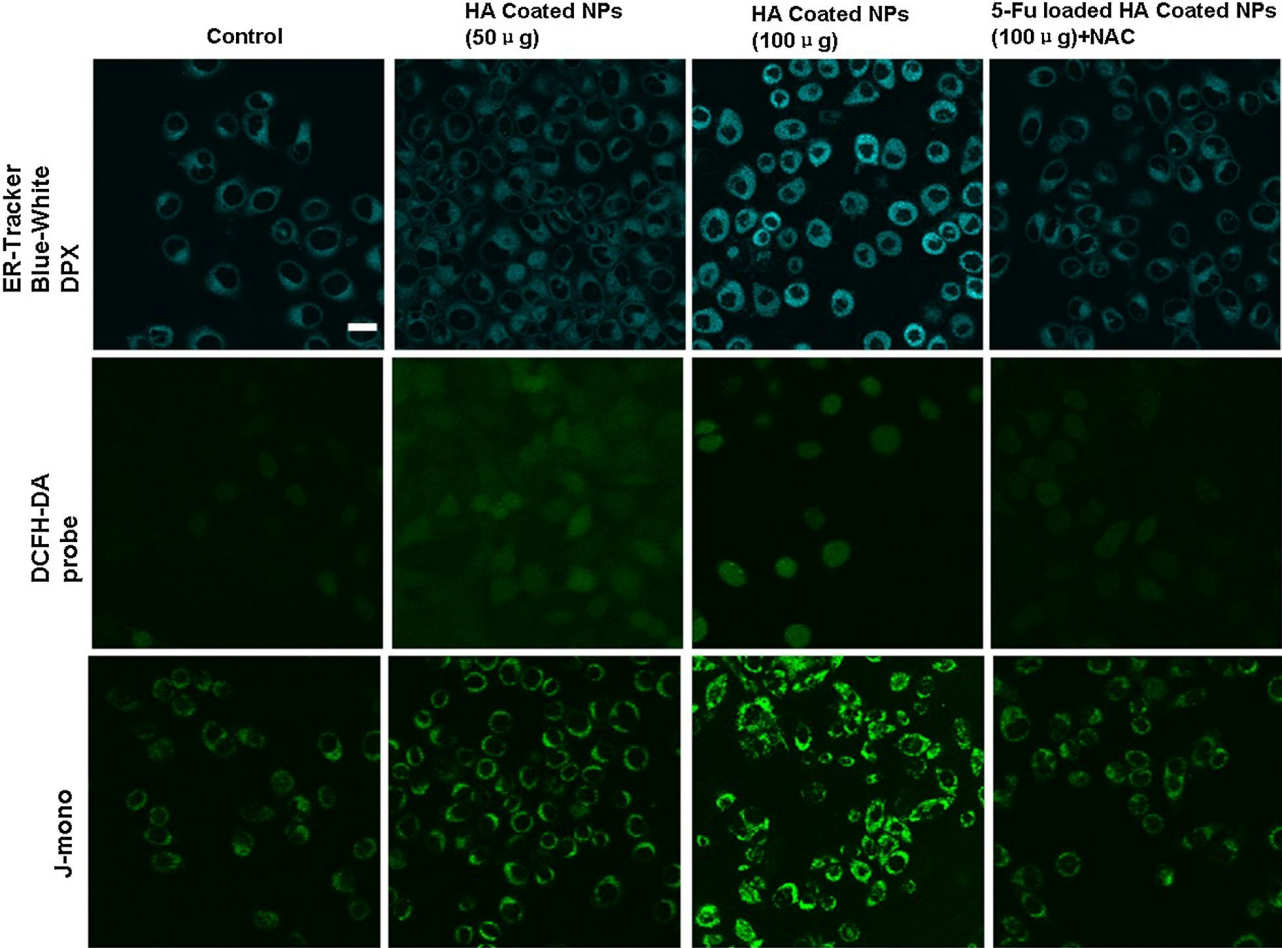
ROS generation in A549 cells treated with HA-coated NPs, ER staining with the ER Tracker blue–white DPX probe, and image changes of the mitochondrial membrane potential following treatment with HA-coated NPs. The *scale bar* in all figure parts is 100 μm

As shown in Fig. 5, we found that HA-coated NPs generated the production of ROS and damaged the integrity of the mitochondria by decreasing the mitochondrial membrane potential. With increasing dosages of NPs, a strong green fluorescence was observed in cells, indicating that NPs accelerated the production of intracellular ROS, showing a dose-dependent relationship. In addition, the intensity of the green fluorescence from the JC-1 monomer continued to increase, suggesting that the mitochondrial membrane potential exhibited a downward trend in a dose-dependent manner. Furthermore, the degree of ER stress was significantly enhanced by the induction of HA-coated NP exposure. However, when given antioxidant NAC to inhibit ROS generation, the mitochondrial membrane potential increased. This finding indicated that HA-coated NPs induced the massive production of oxygen free radicals in cells and damaged the integrity of the mitochondrial membrane by reducing its membrane potential, thus resulting in the activation of the mitochondrial-mediated apoptosis pathway.

### Cell apoptosis and necrosis

**Fig. 6:**
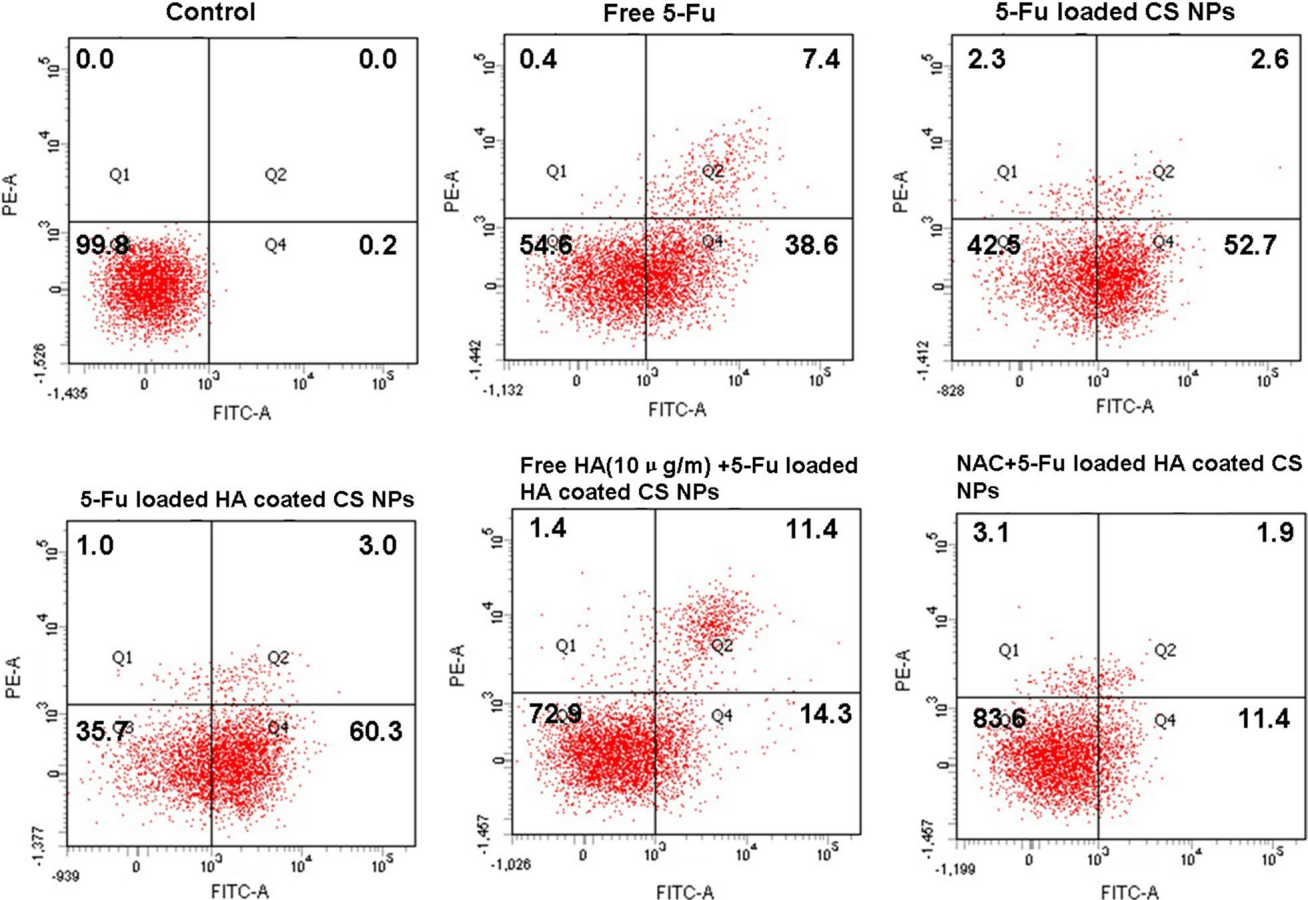
Cell apoptosis determined by Annexin V–fluorescein isothiocyanate/propidium iodide staining. The results were determined after incubation with free 5-Fu, 5-Fu-loaded CS NPs, 5-Fu-loaded HA-coated CS NPs, free HA and 5-Fu-loaded HA-coated CS NPs, and 5-Fu-loaded HA-coated CS NPs combined with NAC for 24 h (n = 3)

When A549 cells were incubated with free 5-Fu, 5-Fu-loaded NPs, and 5-Fu-loaded HA-coated CS NPs, respectively, the ratios of double (Annexin V/PI)-positive cells in A549 cells were analyzed by flow cytometry. As shown in Fig. 6, as compared with free 5-Fu and 5-Fu-loaded uncoated NPs, 5-Fu-loaded HA-coated CS NPs induced the highest apoptosis effects, and the ratio of double (Annexin V/PI)-positive cells in A549 cells was 64.3%. This suggested that HA-coated CS NPs enhanced drug delivery and accumulation, as mediated by HA and CD44; further NP exposure activated the ROS-mediated mitochondrial apoptosis pathway. Therefore, the anti-tumor efficacy of the drug had significantly improved. With the addition of free HA, the internalization of drug-loaded NPs was limited due to the CD44-based binding competition between HA and HA-coated CS NPs; moreover, the apoptosis effects were also decreased, and the ratio of double (Annexin V/PI)-positive cells in the A549 cells was 27.1%. When cells were treated with NAC and 5-Fu-loaded HA-coated CS NPs, the apoptosis effects significantly decreased and the ratio of double (Annexin V/PI)-positive cells in the A549 cells was 16.4%. This may indicate that the addition of NAC inhibited the ROS generation induced by the internalization of NPs, and it further blocked the ROS-mediated mitochondrial apoptosis pathway, thus limiting the induction of apoptosis.

### Western blot analysis

**Fig. 7:**
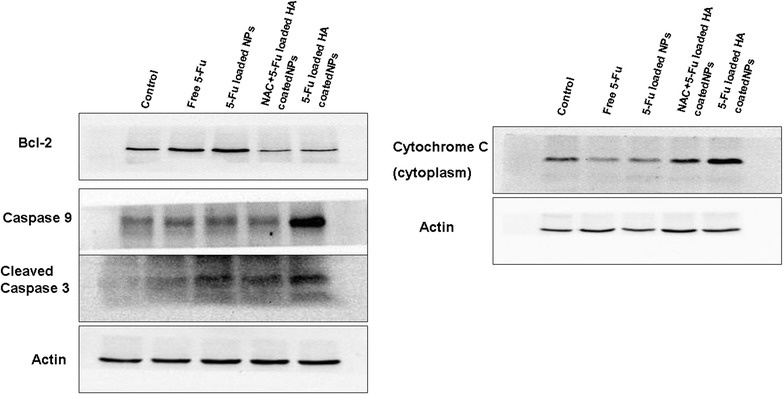
Western blot analyses. The results show the expression levels of cleaved caspase 3, caspase 9, Bcl-2, and cytochrome C in A549 cells treated with free 5-Fu, 5-Fu-loaded CS NPs, 5-Fu-loaded HA-coated CS NPs, and 5-Fu-loaded HA-coated CS NPs combined with NAC

The effects of 5-Fu or 5-Fu-loaded NPs on the mitochondrial apoptosis pathway were investigated by conducting Western blot to examine some mitochondrial apoptosis-related factors such as cytochrome C, caspase precursor, and apoptosis-inducing factors. The results (Fig. 7) showed that compared with free 5-Fu and 5-Fu-uncoated NPs, 5-Fu-loaded HA-coated CS NPs induced the highest apoptosis effects, represented by the highest expression of cytochrome C, caspase-9, and cleaved caspase-3 in A549 cells; the expression level of Bcl-2 was the lowest. This finding highlighted that more 5-Fu-loaded HA-coated CS NPs had accumulated in the cells due to the interaction between HA and CD44, and this led to significant apoptosis. Furthermore, it was observed that cytochrome C in the cytoplasm treated with 5-Fu-loaded HA-coated CS NPs was expressed at the highest level, proving that HA-coated CS NPs damaged the integrity of the mitochondria and decreased its membrane potential, thus resulting in activation of the mitochondrial apoptosis pathway. Following NP exposure, the mitochondrial membrane potential was reduced and cytochrome C was released from the mitochondria to the cytoplasm; furthermore, an important anti-apoptotic protein (Bcl-2) was inhibited and initiated the activation of apoptosis-related proteins such as caspase 9 and 3, eventually aiding in the significant apoptosis of tumor cells. When given the antioxidant NAC, the increased expressions of caspase 9 and cleaved caspase 3 were suppressed and the expression of cytochrome C in the cytoplasm was also reduced. This indicated that NAC decreased the production of ROS generated by the exposure of HA-coated CS NPs and it maintained the integrity of the mitochondrial membrane potential. Therefore, the ability to transfer cytochrome C from the mitochondria to the cytoplasm was blocked and the ROS-mediated mitochondrial apoptosis pathway was further inhibited.

## Discussion

The important physiological function of mitochondria is to produce adenosine triphosphate (ATP) by oxidative phosphorylation; they are also involved in apoptosis and they regulate calcium levels in the cytoplasm and mitochondria [26, 27]. It was found that the occurrence and development of tumorigenesis were associated with disordered mitochondrial function. The integrity of the mitochondria was more susceptible to damage by the induction of ROS [28], thus leading to difficulties in transcription and in the synthesis of related peptides, further triggering the mitochondrial-mediated apoptotic pathway. Therefore, researchers managed to interfere with the integrity and function of the mitochondria to promote cell death. NPs, as a nanoscale carrier, showed excellent value and potential for improving drug delivery. As their particle sizes range from 10 to 500 nm, drug-loaded NPs can be selectively retained at the tumor site (known as the EPR effect), and they can conjugate specific targeting molecules at the NPs’ surface to achieve active targeted therapy by binding with specific cell-surface receptors [29, 30]. In addition, the internalization of some NPs could specifically occur at the mitochondria, thus increasing ROS generation and enhancing synergistic antitumor efficacy by activating mitochondrial-meditated apoptosis [31, 32]. In our study, we also found that HA presented as an active targeting factor and was absorbed at the surface of the CS NPs; these NPs bound to specific CD44 receptors on the CD44-overexpressed tumor cell surface, thus improving the targeting efficiency of NPs and accelerating drug accumulation within the cells. Importantly, HA-coated CS NPs were distributed in the mitochondria and they generated the massive production of ROS and activated ROS-induced mitochondrial disorders. This suggested that compared with free 5-Fu and 5-Fu-loaded uncoated CS NPs, 5-Fu-loaded HA-coated CS NPs enhanced CD44 receptor-mediated endocytosis and led to increased intracellular uptake of the drug. Furthermore, HA-coated CS NPs also generated the massive production of ROS and activated the mitochondrial apoptotic pathway, thus enhancing the drug’s synergistic antitumor effects.

## Conclusions

Biocompatible and biodegradable HA-coated CS NPs were developed to encapsulate a chemotherapeutic drug (5-Fu) to enhance drug accumulation in tumor cells and to improve the drug’s antitumor efficiency by achieving targeted drug delivery via CD44. The results showed that the size of the 5-Fu-loaded HA-coated CS NPs was smaller and more homogenously distributed; moreover, the morphology of NPs was subspheroidal and their zeta potential was positive and valued at 15.6 ± 3.7 mV. It is important to note that 5-Fu loaded HA-coated CS NPs showed a sustained and biphasic release profile, as 75% of the total 5-Fu was released in 48 h. We found that compared with free drugs and uncoated NPs, particularly given the interaction between HA and CD44, HA-coated NPs enhanced drug accumulation by effectively transporting NPs into CD44-overexpressed tumor cells and inducing cell apoptosis. Exposure of 5-Fu-loaded HA-coated NPs enhanced the generation of ROS and resulted in ROS-induced disorders in mitochondrial function, further activating the mitochondrial-mediated apoptosis pathway.

## Abbreviations

HA: hyaluronic acid
CS: chitosan
NPs: nanoparticles
ROS: reactive oxygen species
